# Supplementary material for: Muscle‐derived GDF15 drives diurnal anorexia and systemic metabolic remodeling during mitochondrial stress
Source: EMBO Rep. 2020 Feb 6;21(3):e48804. doi: 10.15252/embr.201948804 (PMC7054681; doi:10.15252/embr.201948804)
Supplement: Supplementary file 1 — Expanded View Figures PDF [file EMBR-21-e48804-s001.pdf]

Expanded View Figures

Figure EV1. Skeletal muscle phenotype and function of male *Gdf15*-KO animals.

A Plasma GDF15 levels at 20 weeks of age (WT n = 9, KO n = 9).
B Voluntary wheel running (VWR) activity shown hourly over 24 h at 15 weeks of age (n = 4 per genotype).
C Grip strength at 10–20 weeks of age (WT n = 15, KO n = 14).
D Skeletal muscle mass relative to body lean mass of quadriceps (Quad), gastrocnemius (Gastroc), soleus, and EDL (WT n = 9, KO = 10).
E Representative H&E histological staining of tibialis anterior (TA) muscle (G) (scale bars represent 50 μm)
F–H Mitochondrial respiratory capacity (oxygen consumption rate, OCR) from oxidative soleus (SOL) (F) and glycolytic extensor digitorum longus (EDL) muscle fibers (G), and NetOXPHOS control ratio (H) (n = 5 per genotype).
I, J Representative immunoblots of OXPHOS in quadriceps (Quad) muscle (I) and their corresponding quantifications normalized to MFN2 (J) (n = 6 per genotype).
K Skeletal muscle (Quad) relative mRNA expression of ISR components (WT n = 8, KO n = 6).
L Representative immunoblots of ISR component eIF2α and phospho-eIF2α (p-eIF2a^{Ser51}), TG sample was included as positive control.
M Skeletal muscle (Gastroc) enzyme activity of NQO1 and GPX (WT n = 8, KO n = 6).

Data information: Data shown are from male wild-type (WT) versus *Gdf15*-KO (KO) mice. Circulating plasma parameters are expressed as interleaved box and whiskers (min to max) plots, and all other data are expressed as means ± SEM; *P*-value calculated by unpaired Student's *t*-test.

© 2020 The Authors

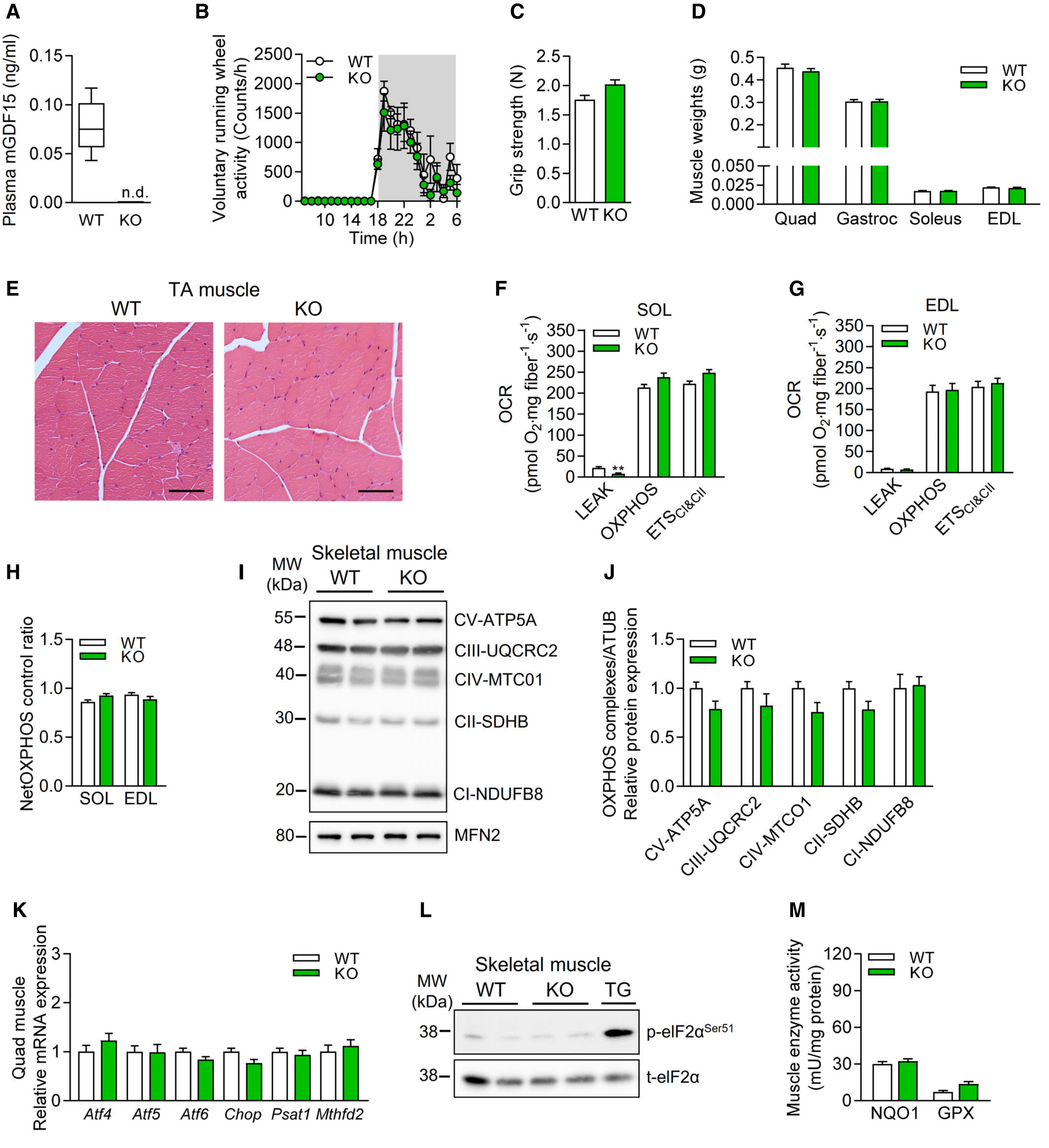

Figure EV1.

© 2020 The Authors

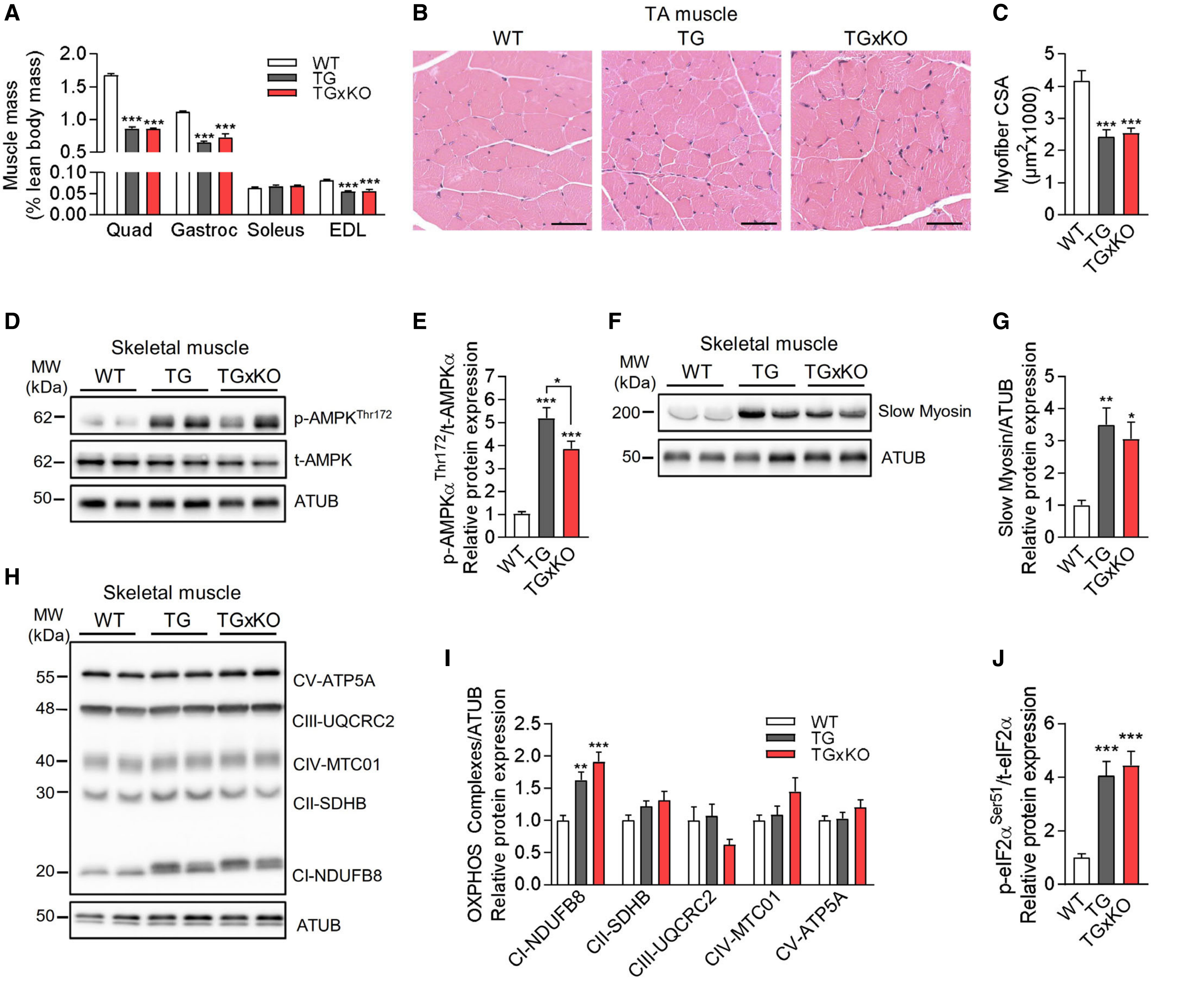

Figure EV2. GDF15-independent muscle wasting and cell-autonomous stress response.

- A Muscle mass relative to body lean mass of quadriceps (Quad), gastrocnemius (Gastroc), soleus, and extensor digitorum longus (EDL) muscles in 20-week-old male mice (WT *n* = 9, TG, *n* = 9, TGxKO = 10).
- B, C Representative H&E histological staining of tibialis anterior (TA) muscle (B), and cross-sectional area (CSA) of myofibers (C) at 20 weeks (WT *n* = 8, TG *n* = 6, TGxKO *n* = 8), scale bars represent 50 µm.
- D–I Representative immunoblots and their corresponding quantifications (WT *n* = 8, TG *n* = 8, TGxKO *n* = 8), normalized to ATUB of p-AMPK^{Thr172}/t-AMPK (D, E), slow myosin (F, G), and OXPHOS (H, I) in quadriceps (Quad) muscle at 20 weeks of age.
- J Quantification of ISR component eIF2α and of phospho-eIF2α (p-eIF2a^{Ser51}) relative protein expression in quadriceps skeletal muscle at 95 weeks of age (WT *n* = 8, TG *n* = 8, TGxKO *n* = 8).

Data information: Data shown are from male wild-type (WT), *Ucp1*-TG (TG), and *Ucp1*-TGx*Gdf15*-KO (TGxKO) mice. Data are expressed as means ± SEM; *P*-value calculated by one-way ANOVA with Tukey's post hoc test; **P* < 0.05, ***P* < 0.01, ****P* < 0.001.

© 2020 The Authors

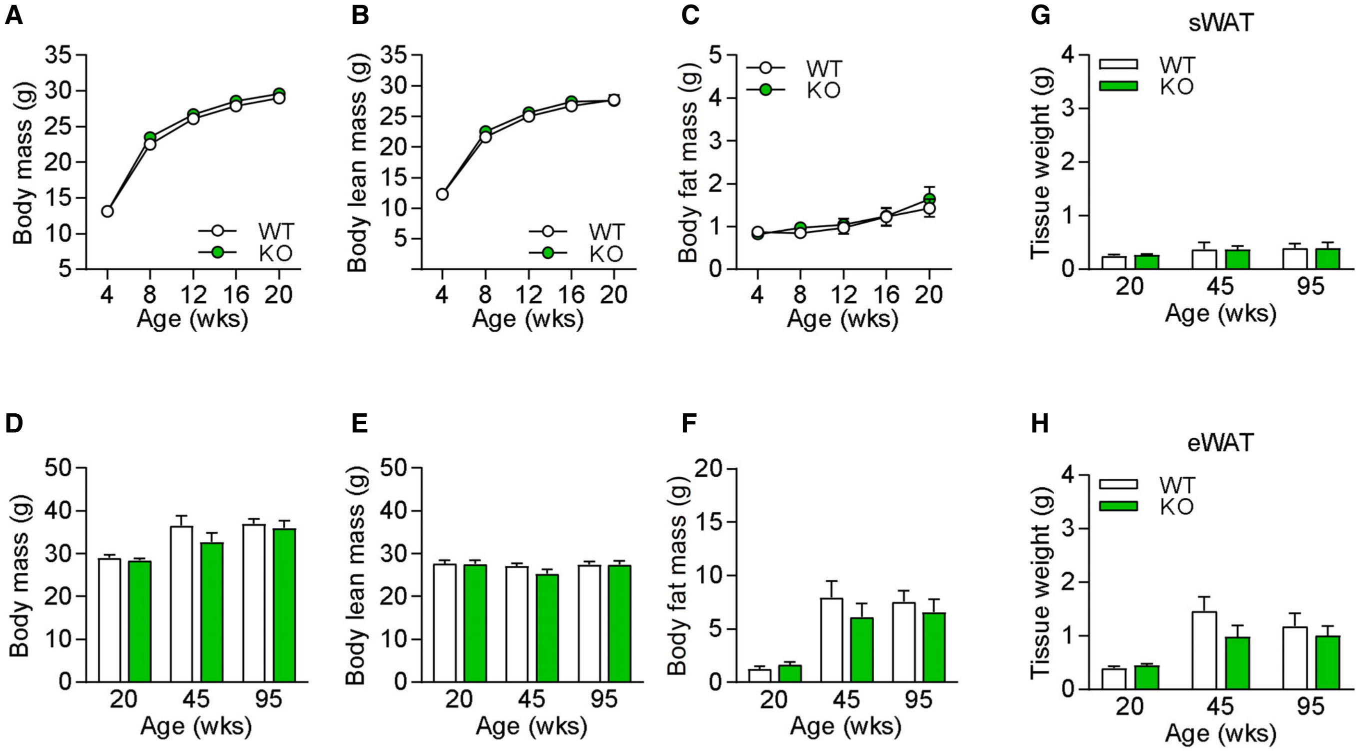

Figure EV3. Body composition and white adipose tissue mass of male *Gdf15*-KO animals.

A–C Body mass (A), body lean mass (B), and body fat mass (C) development.

D–F Body mass (D), body lean mass (E), and body fat mass (F) during aging at 20, 45, and 95 weeks of age.

G, H Subcutaneous white adipose tissue (sWAT) (G) and epididymal white adipose tissue (eWAT) (H) mass development at 20, 45, and 95 weeks of age.

Data information: All data are from male wild-type (WT) versus *Gdf15*-KO (KO) mice at 20 weeks (WT n = 10, KO n = 10), 45 weeks (WT n = 5, KO n = 5), and 95 weeks (WT n = 8, KO n = 6). All data are expressed as means ± SEM; P-value calculated by unpaired Student's t-test.

 © 2020 The Authors

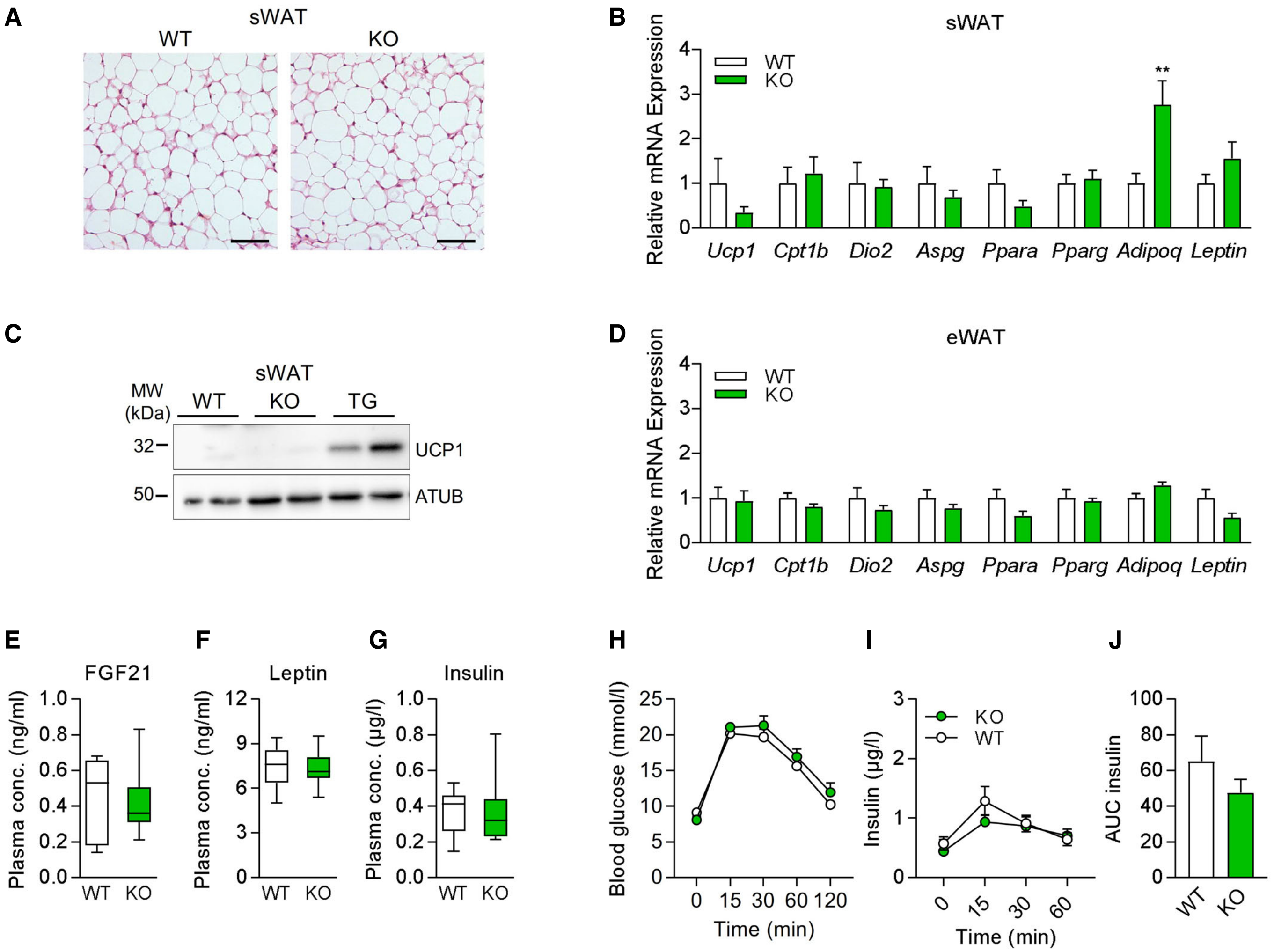

Figure EV4. White adipose tissue browning profile, plasma metabolic mediators, and insulin sensitivity of male *Gdf15*-KO animals.

A, B Representative H&E histological staining of sWAT at 20 weeks of age (scale bars represent 50 μm) (A) and relative mRNA expression profile in sWAT of male mice (WT *n* = 7, KO *n* = 8) (B).

C Representative immunoblots of UCP1 protein expression in sWAT of male mice at 20 weeks of age. TG samples were included as positive control.

D Relative mRNA expression profile in eWAT (WT *n* = 7, KO *n* = 8).

E Plasma FGF21 levels from male mice at 20 weeks of age (WT *n* = 8, KO *n* = 10).

F Plasma Leptin levels from male mice at 20 weeks of age (WT *n* = 8, KO *n* = 10).

G Post-absorptive plasma insulin levels at 20 weeks of age (WT *n* = 8, KO *n* = 10).

H–J Blood glucose (H) and insulin levels (I) with total area under the curve (AUC) of insulin (J) during oral glucose tolerance test (oGTT) at 17 weeks of age (WT *n* = 5, KO *n* = 3).

Data information: All data are from male wild-type (WT) versus *Gdf15*-KO (KO) mice at 20 weeks. Circulating plasma parameters are expressed as interleaved box and whiskers (min to max) plots, and all other data are expressed as means ± SEM; *P*-value calculated by unpaired Student's *t*-test.

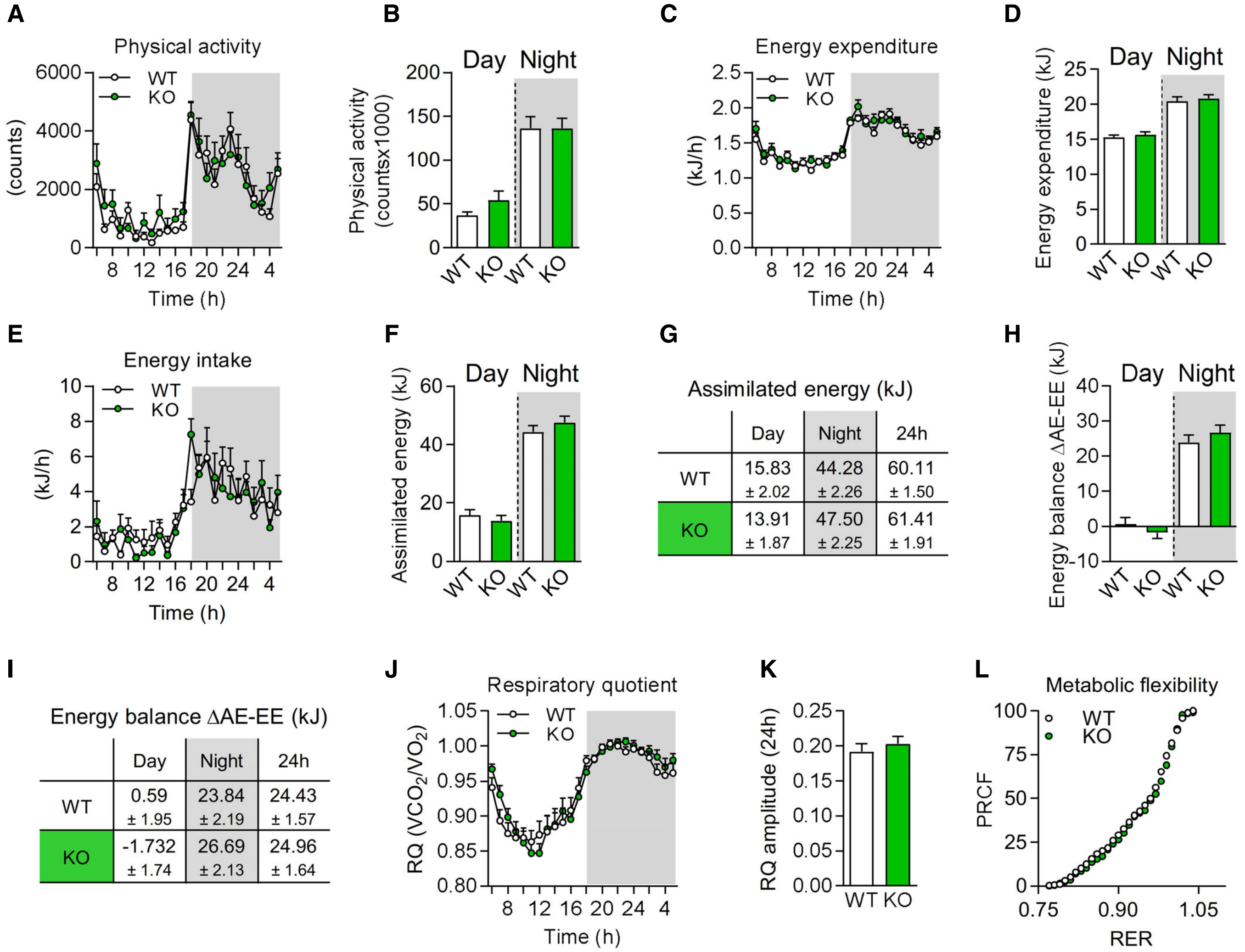

G — Assimilated energy (kJ)

	Day	Night	24h
WT	15.83 ± 2.02	44.28 ± 2.26	60.11 ± 1.50
KO	13.91 ± 1.87	47.50 ± 2.25	61.41 ± 1.91

I — Energy balance ΔAE-EE (kJ)

	Day	Night	24h
WT	0.59 ± 1.95	23.84 ± 2.19	24.43 ± 1.57
KO	-1.732 ± 1.74	26.69 ± 2.13	24.96 ± 1.64

Figure EV5. In vivo metabolic phenotyping of male *Gdf15*-KO animals.

A–F Physical activity (A, B), energy expenditure (C, D), total assimilated energy (E, F) of male mice shown hourly over 24 h and as day and night time.
G Table showing mean values of assimilated energy (kJ) at day/night time and per 24 h.
H Energy balance calculated as delta of assimilated energy (AE) and energy expenditure (EE) and energy balance (J) per 24 h.
I Table showing mean values of energy balance (ΔAE-EE, kJ) at day/night time and per 24 h.
J–L Respiratory quotient (RQ) shown hourly over 24 h (J), RQ amplitude (K), and metabolic flexibility via percentage relative cumulative frequency (PRCF) (L).

Data information: All data are from male wild-type (WT) versus *Gdf15*-KO (KO) mice at 17–18 weeks of age (WT n = 12, KO n = 11) and shown as means ± SEM; *P*-value calculated by unpaired Student's *t*-test.

 © 2020 The Authors
